# A systematic review and meta-analysis of intraperitoneal anastomosis versus extraperitoneal anastomosis in laparoscopic left colectomy

**DOI:** 10.3389/fonc.2024.1464758

**Published:** 2024-09-27

**Authors:** Wenjie Zhou, Xueting Wang, Jie Dan, Mingjie Zhu, Ming Li, Ke Liu, Qian Liao, Yonghong Wang

**Affiliations:** ^1^ Department of Gastrointestinal Surgery, The People’s Hospital of Leshan, Leshan, Sichuan, China; ^2^ Department of Scientific Research and Teaching, The People’s Hospital of Leshan, Leshan, Sichuan, China

**Keywords:** laparoscopic left colectomy, intraperitoneal anastomosis, extraperitoneal anastomosis, postoperative complications, meta-analysis

## Abstract

**Background:**

The effectiveness of the anastomosis method for laparoscopic left colectomy (LLC) remains inconclusive. Thus, a systematic review and meta-analysis were conducted to compare the outcomes between intraperitoneal anastomosis (IPA) and extraperitoneal anastomosis(EPA)in LLC.

**Methods:**

PubMed, Embase, the Cochrane Library, CNKI, and WanFangData were systematically searched for relevant literature. The literature was screened independently by two groups, and data were extracted and evaluated for bias. Meta-analysis was performed using Revman5.4 software.

**Results:**

Twelve studies with a total of 1,278 patients were included in our meta-analysis. Compared with the EPA group, the IPA group had less blood loss [odds ratio (OR)=–20.32, 95% confidence interval (CI) (−27.98–12.65), p<0.00001], a lower overall complication rate [OR=0.45, 95% CI (0.33–0.63), p<0.00001], fewer non-severe complications [OR=0.44, 95% CI (0.30–0.64), p<0.0001], and fewer surgical site infections [OR=0.39, 95% CI (0.21–0.71), p=0.002]. Additionally, a longer operation time appeared in the multicenter and propensity score matching (PSM) subgroups of the IPA group. Furthermore, patients in the IPA group had an earlier exhaust time and shorter hospital stays. There were no significant differences between the two groups regarding severe complications, anastomose-related complications, postoperative blood transfusion, ileus, reoperation rate, time to stool, pathologic sample length, and lymph node dissection number.

**Conclusion:**

IPA seems more advantageous than EPA for patients receiving LCC in terms of complications and postoperative recovery and has similar oncological outcomes. However, it may take longer and be more difficult to perform.

**Systematic Review Registration:**

https://www.crd.york.ac.uk/PROSPERO/#recordDetails PROSPERO, identifier (CRD4202454391).

## 1 Introduction

Colorectal cancer is one of the most common malignancies, with the third- and second-highest morbidity and mortality rates worldwide, respectively, in 2022 (1). At present, laparoscopic techniques and complete mesocolon excision have become the preferred methods for the radical resection of colon cancer (2, 3). However, LLC is a special radical operation for colon cancer. Colon-to-colon anastomosis after specimen removal, including the partial or total removal of the left colon and left transverse colon, is technically demanding. At present, there are four hotspots in surgical research (4–7): splenic flexure mobilization, lymph node dissection, digestive tract reconstruction, and robotic surgery. Generally, LLC is usually performed with EPA, which is actually the laparoscopically assisted left colectomy (8). However, in obese patients or patients with short mesocolons and muscular abdominal walls, EPA may lead to sizeable abdominal wall incisions, significant postoperative pain, and a high incidence of incision infection and incisional hernia (9–12). To achieve tension-free anastomosis (13), more intestines need to be mobilized or the length of the bowel resection should be reduced. IPA seems to overcome these shortcomings but there is also the chance of tumor cells or intestinal fluid spilling into the abdominal cavity. Additionally, IPA is more complex and time-consuming and is probably associated with an increasing risk of anastomotic leakage, abdominal infection, and anastomotic stenosis after surgery.

The original published studies are mainly single-arm, small-sample cohort, or experience-sharing studies. Therefore, we conducted a systematic review and meta-analysis comparing the outcomes of IPA and EPA with a large number of patients receiving LLC.

## 2 Methods

This study was conducted according to the current preferred reporting items for systematic reviews and meta-analyses (PRISMA) (14) and the methodological quality guidelines for systematic reviews (AMSTER) and has been registered in PROSPERO (registration number CRD42024543918).

### 2.1 Search strategy

PubMed, Embase, the Cochrane Library, CNKI, and WanFangDATA were systematically searched by two researchers for articles published independently. No language restrictions were applied, and our search also included all references of all articles, which were retrieved in full text. The retrieval scheme adopted the method of combining subject words with free words. The search keywords were “colectomy”, “anastomosis”, and “left” and were limited to the title and abstract. The last literature search was on 1 May 2024. Two researchers independently screened the retrieved literature and assessed the eligibility of each selected study included in the meta-analysis. Differences should be resolved through consensus and, if necessary, through meetings.

### 2.2 Inclusion and exclusion criteria

The inclusion criteria were as follows: (1) All original studies using colon-to-colonic anastomosis in laparoscopic or robotic left colectomy, either prospective or retrospective; (2) the surgical interventions were IPA and EPA, regardless of stapling or manual anastomosis; (3) outcome indicators must have reports involving complications; and (4) a Newcastle-Ottawa Scale (NOS) score of more than five for cohort studies. The most recent study was selected for inclusion when duplicate or overlapping articles were published by the same institution and researcher.

The exclusion criteria were as follows: (1) an end-to-end colonic anastomosis with a circular stapler through the anus and (2) conference abstracts that did not provide data on complications or reports with incomplete data.

### 2.3 Data extraction

Two researchers independently extracted literature data. When there were differences, they were verified by a third researcher, and the data from the final analysis were discussed and determined. The data we extracted were as follows: (1) general literature data, including the first researcher name, journal, publication year, country or region, study type, propensity score, multicenter clinical study, case enrollment time, follow-up time, and sample size; (2) basic patient information including age, body mass index (BMI), gender, lesion site, the tumor node metastasis classification (TNM), and the American College of Anesthesiologists score (ASA); (3) perioperative protocols and surgical details including preoperative bowel preparation and antibiotic use, enhanced recovery after surgery (ERAS), anastomosis plan, stapler, specimen length, incision site and length, operation time, blood loss volume, intraoperative accident, number of lymph node dissections, and specimen length; and (4) postoperative recovery data, including the time to flatus and stool, the pain score, analgesic drug use, the length of hospital stays, postoperative complications, recurrence, and survival analysis results. If important information was missing, the corresponding researcher was contacted if possible. The primary outcomes of this study were information on surgical difficulty and postoperative complications. Secondary outcomes included postoperative recovery and oncological outcomes. For continuous data with quartile or median and extreme values, mean and standard deviation were extracted according to the methods of D. Luo, J. Shi, and X. Wan, and valid data that could not be extracted were not included in the meta-analysis (15–18).

### 2.4 Quality assessment

Two researchers independently used the Newcastle-Ottawa quality assessment tool (NOS scale) to evaluate the quality of the literature, and any differences were resolved through consensus. Eight projects were rated for quality in three areas: selection (up to 4 points), comparability (up to 2 points), and outcome (up to 3 points). The higher the score, the higher the quality.

### 2.5 Statistical analysis

Statistical analysis was conducted using the Revman5.4 version software provided by the Cochrane Library. Odds ratios (ORs) and mean difference (MD) and their corresponding 95% confidence intervals (95% CI) were calculated for counting and measurement data, respectively. Q test and I^2^ test analyses were used to examine the heterogeneity of the literature. If heterogeneity was high (I^2^ >50%), pooled estimates were calculated using the random-effects method. Otherwise, a fixed random-effects model was used. For cases with high heterogeneity, one-way sensitivity and subgroup analysis were used to explore sources of heterogeneity. To test the stability of meta-analyses, subgroup analyses were performed according to center (multicenter vs. single center), sample size (more than median vs less than median), race (yellow vs. white), propensity score matching (PSM), study type (prospective vs. retrospective), and time to publication (earlier than median time vs. later than median time). A funnel plot was used to test publication bias for more than ten included studies. A p-value <0.05 for pooled data was significant.

## 3 Results

### 3.1 Study characteristics

**Figure 1:**
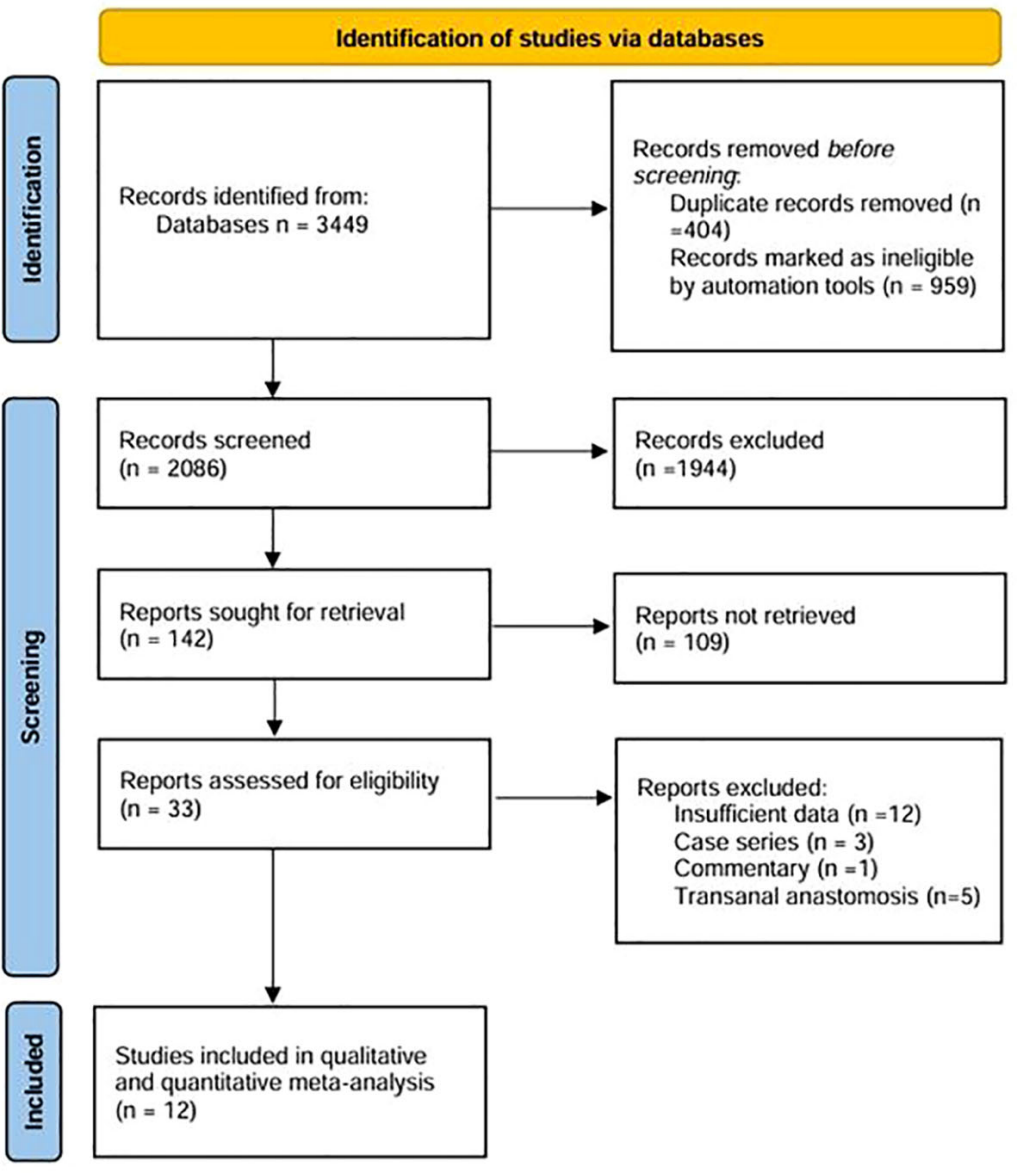
The PRISMA diagram for the selection of the studies.

According to the initial search strategy, 4,077 articles were retrieved, 12 of which, with a total of 1,278 cases (524 cases in the IPA group and 754 cases in the EPA group), met the inclusion criteria. No cases of robotic surgery have been reported in the literature. There were three prospective studies and nine retrospective studies. Four studies used PSM, five were multicenter clinical studies, and the rest were single-center studies. Four were followed up in the medium and long term; the others were followed up in the short term. Nine studies were from Asia and three were from Europe. In one study that included left and right colectomy, the meta-analysis only extracted data from the left colectomy subgroup. The literature screening process and data characteristics are described in detail in **Figure 1** and **Tables 1**–**3**. **Appendix 1** shows the different types of anastomosis.

**Table 1:** The general characteristics and NOS scores of each study.

First author	Year	Case enrollment period	Origin	Follow-up time	Study type	Number of centers	PSM	NOS
**Zhang, M. (** 19 **)**	2024	December 2014 to December 2019	China	100 months	Retrospective	3	Yes	8
**Teramura, K. (** 20 **)**	2023	January 2018 to June 2021	Japan	30 days	Retrospective	1	Yes	8
**Guo, Y. (** 21 **)**	2023	January 2015 to September 2021	China	30 days	Retrospective	2	Yes	8
**Zhong, H. (** 13 **)**	2023	2019 to 2021	China	30 days	Retrospective	7	No	7
**Wang, L. (** 22 **)**	2022	July 2016 to September 2019	Taiwan	At least 2 years	Retrospective	1	No	7
**Ge, L. (** 23 **)**	2020	October 2017 to February 2019	China	NA	Retrospective	1	No	6
**Grieco, M. (** 24 **)**	2019	January 2008 to August 2017	Italy	From discharge to Dec 31, 2017	Retrospective	3	Yes	8
**Zhong, K. (** 25 **)**	2019	May 2014 to October 2017	China	NA	Prospective	1	No	6
**Wang, N. (** 26 **)**	2018	Control group January 2015 to July 2016 Experimental group August 2016 to August 2017	China	NA	Retrospective	1	No	6
**Milone, M. (** 27 **)**	2018	January 2005 to December 2015	Italy	30 days	prospective	5	No	7
**Swaid, F. (** 28)	2016	January 2005 to September 2014,	Israel	30 days	Retrospective	1	No	7
**Carlini, M. (** 29 **)**	2016	January 2004 to October 2015,	Italy	18 to 95 months	Prospective	1	No	6

PSM, propensity Score Match; NOS, Newcastle-Ottawa Scale.

NA, Not Available.

**Table 2:** The general characteristics of the study population.

Author and year	Sample size	Groups	Gender (M/F)	Age	BMI	ASA I-II/≥III	Lesion location	TNM stage (0/I/II/III/IV)
**Zhang, M. (** 19) **2024**	258	IPA	84/45	56.7 (22.0–93.0)	24.5 (16.7–38.4)	NA	Splenic flexure	0/22/46/61/0
		EPA	83/46	57.4 (24.0–86.0)	24.3 (16.6–33.6)	NA		0/20/50/59/0
**Teramura, K. (** 20) **2023**	43	IPA	NA	NA	NA	NA	Left colon	NA
		EPA	NA	NA	NA	NA		NA
**Guo, Y. (** 21 **). 2023**	225	IPA	58/26	62 (54, 69)	23.8 (22.1, 25.8)	80/4	Proximal 1/3 of the sigmoid colon, Descending colon, Distal 1/3 of the transverse colon to splenic flexure	NA
		EPA	99/42	61 (54, 69)	23.5 (21.8, 26.7)	131/10		NA
**Zhong, H. (** 13 **). 2023**	134	IPA	7/13	58.1 ± 11.4	22.9 ± 3.4	19/1	Transverse colon	0/1/10/9/0
		EPA	70/44	60.2 ± 12.5	24.1 ± 3.2	108/6		0/7/51/56/0
**Wang, L. (** 22 **). 2022**	117	IPA	23/17	61.45 ± 11.9	23.92 ± 3.1	15/25	Transverse colon, splenic flexure, Descending colon,	2/9/18/11/0
		EPA	45/32	62.65 ± 13.5	23.94 ± 4.6	34/43		0/21/29/27/0
**Ge, L. (** 23) **2020**	86	IPA	19/6	56.8 ± 9.1	24.8 ± 3.1	24/1	Descending colon, Junction of descending colon and sigmoid colon	0/4/10/11/0
		EPA	41/20	54.9 ± 12.0	24.6 ± 3.6	57/4		0/22/24/15/0
**Grieco, M. (** 24 **). 2019**	72	IPA	19/17	71.4 ± 9.9	25.3 ± 4.0	25/11	Splenic flexure	1/10/15/10/0
		EPA	23/13	68.7 ± 6.7	26.0 ± 4.5	28/8		1/7/12/16/0
**Zhong, K. (** 25) **2019**	53	IPA	5/14	62.1 ± 11.9	NA	17/2	Splenic flexure, or descending and sigmoid colon	0/1/8/9/1
		EPA	8/26	61.8 ± 10.3	NA	26/8		0/0/13/19/2
**Wang, N. (** 26) **2018**	37	IPA	10/6	66.4 ± 4.8	23.6 ± 2.3	NA	Distal transverse colon, splenic flexure, descending colon, proximal sigmoid colon	0/0/9/7/0
		EPA	12/9	65.9 ± 5.8	22.7 ± 2.8	NA		0/0/12/9/0
**Milone, M. (** 27 **). 2018**	181	IPA	54/38	66 ± 10.9	29.5 ± 4.3	51/41	Splenic flexure	NA
		EPA	47/42	68.7 ± 10.24	24.7 ± 4.2	51/38		NA
**Swaid, F. (** 28) **2016**	52	IPA	22/11	64.2 ± 12.4	25.4 ± 3.9	26/7	The distal transverse colon, splenic flexure, or descending colon	NA
		EPA	8/11	72.7 ± 2.1	25 ± 3.6	12/7		NA
**Carlini, M. (** 29 **) 2016**	20	IPA	NA	NA	NA	18/2	Splenic flexure	0/8/0/12/0
		EPA	NA	NA	NA	NA		NA

ASA, American Standards Association; BMI, Body Mass Index; TNM, tumor-node-metastasis.

NA, Not Available.

**Table 3:** Surgical details and perioperative characteristics of each study.

Author year	Groups	Sample size	stapled/ hand sewn	Type of anastomosis	Site of extraction	length of incision	ERAS	Bowel preparation	Prophylactic antibiotics	Number of trocars/ ports, (number*size)
**Zhang, M.** (19) **2024**	IA	129	129/0	①iso-peristaltic	Pfannenstiel	5.6 (4.0–10.0)*	NA	mechanical	YES	5, NA
	EA	129	NA	NA	Vertical periumbilical	6.8 (4.0–15.0)*				
**Teramura, K.** (20) **2023**	IA	21	21/0	①	Pfannenstiel, umbilical midline	NA	NA	NA	NA	5, 2*12mm+3*5mm
	EA	22	22/0	①	Umbilical midline	NA				
**Guo, Y.** (21) **2023**	IA	84	83/1	①	Longitudinal midline, off-midline	NA	YES	mechanical, Selective oral antibiotics	YES	NA
	EA	141	104/19	①②③	Longitudinal midline, off-midline	NA				
**Zhong, H.**(13) **2023**	IA	20	20/0		Median periumbilical, Pfannenstiel	4.5 (2.5–6.5)**	NA	NA	NA	NA
	EA	114	99/15	①②	Median periumbilical, Left paramedian	7.5 (4.0–11.0)**				
**Wang, L.** (22) **2022**	IA	40	NA	①③	Pfannenstiel, Midline, Natural orifice specimen extraction, Off-midline	NA	NA	mechanical	YES	4, 2*12mm+2*5mm
	EA	77	NA	①③	Midline, umbilical wound	NA				
**Ge, L.** (23) **2020**	IA	25	25/0	Overlap, delta-shaped	Pfannenstiel	4.2±2.2	NA	mechanical	YES	5, 2*12mm+3*5mm
	EA	61	61/0	③	Left transrectus abdominis	7±2.5				
**Grieco, M.** (24) **2019**	IA	36	36/0	①	Pfannenstiel	5.2±0.6	YES	NA	YES	3-5, 2*12mm+(1-3)*5mm
	EA	36	15/21	①,③	Left subcostal	13.3±2.3				
**Zhong,K.** (25) **2019**	IA	19	19/0	①overlap	Periumbilical	NA	NA	NA	NA	4
	EA	34	34/0	②	Extended left punctured hole	NA				
**Wang, N.** (26) **2018**	IA	16	16/0	①overlap	Periumbilical, Pfannenstiel	3.9±0.9	NA	NA	NA	5, (2*12mm+3*5mm) or (3*12mm+2*5mm)
	EA	21	21/0	FEEA	Left transrectus abdominis	6.9±0.3				2*12mm+3*5mm
**Milone, M.** (27) **2018**	IA	92	NA	①③	Mini-Pfannenstiel	7.8±1.3	YES	NA	NA	NA
	EA	89	NA	①③	Mini-laparotomy midline	9.5±3.1				
**Swaid, F.** (28) **2016**	IA	33	33/0	①iso-peristaltic	Mini-Pfannenstiel	5.8±0.9	NA	NA	YES	4, 2*12mm+2*5mm
	EA	19	19/0	①iso-peristaltic	Left off-midline	8.2±0.9				
**Carlini, M.** (29) **2016**	IA	9	9/0	①	Pfannenstiel	NA	NA	NA	NA	NA
	EA	11	0/11	①	Off-midline	NA				

①:Side-to-Side; ②:Side-to-End, ③:End-to-End ; FEEA: Functional End-to-End Anastomosis; ERAS: Enhanced Recovery After Surgery

Values are presented as mean±standard deviations, * as mean and range (min-max values), ** as median and range (min-max values).

NA, Not Available

### 3.2 Quality assessment

All the 12 articles included were prospective cohort studies or retrospective cohort studies. Therefore, the NOS scale was used to evaluate the quality of the article. The quality of the article was evaluated from eight aspects. Article scores are shown in **Table 1**. All articles had an NOS score of more than 5. Therefore, the overall quality of these 12 articles was considered to be good.

### 3.3 Meta-analysis results

#### 3.3.1 Difficulty of the operations

##### 3.3.1.1 Operation time

**Figure 2:**
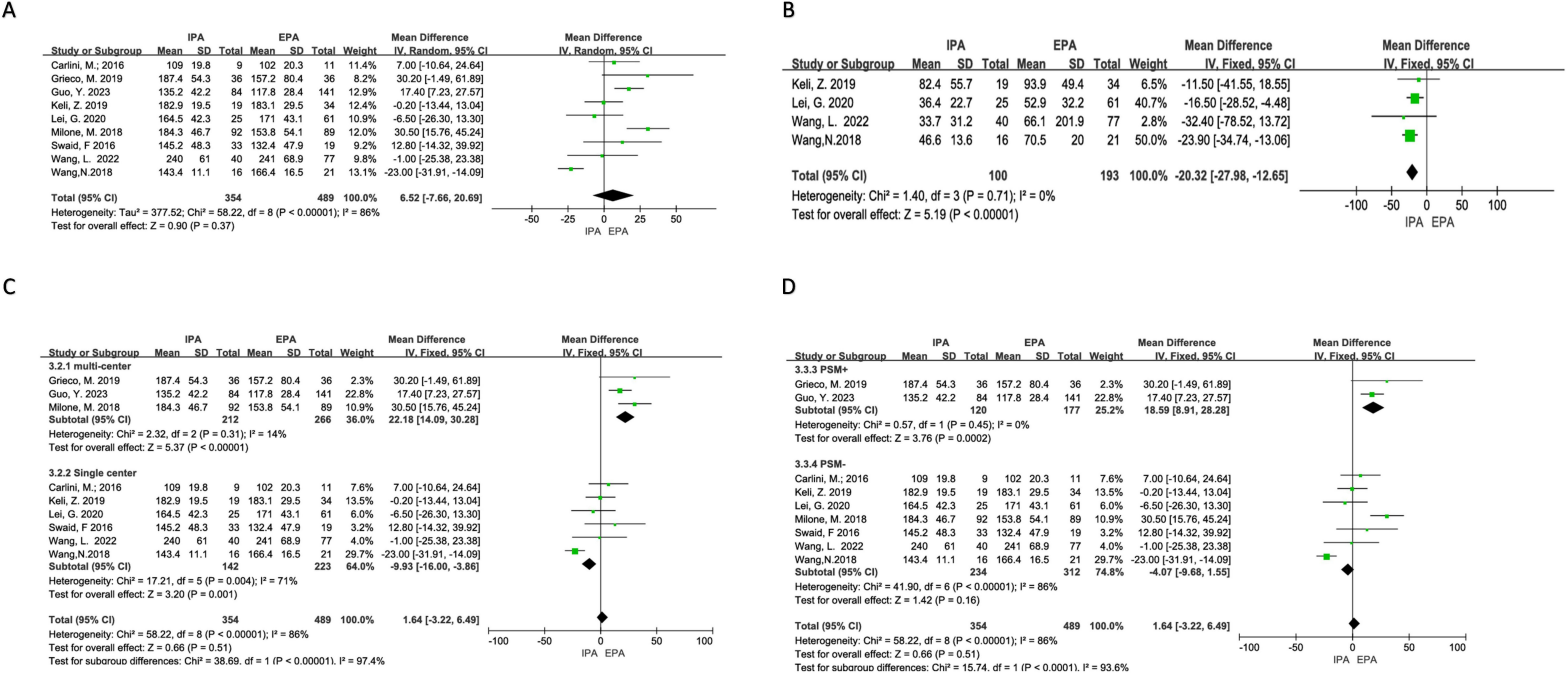
Difficulty of the operations. **(A)** Operation time; **(B)** Blood loss; **(C)** Subgroup analysis of operation time according to the number of research centers; **(D)** Subgroup analysis of operation time according to PSm.

Nine studies were included in the analysis, and there was apparent heterogeneity (I^2^ = 86%, χ^2^test p<0.00001). Sensitivity analysis could not find the source of heterogeneity. Therefore, a random-effects model was used. The results showed no difference in the operation time between IPA and EPA [OR=6.52, 95% CI (−7.66–20.7), p=0.37]. Subgroup analysis suggested that the multicenter PSM subgroups had low heterogeneity. In these subgroups, the operation time of IPA was longer than that of EPA [multicenter subgroup I^2^ = 14%, χ^2^p=0.31 OR=22.18, 95% CI (14.09–30.28), p<0.00001], [PSM+ subgroup I^2^ = 0%, χ^2^p=0.45 OR=18.59, 95% CI (8.91–28.28), p=0.0002] (**Figure 2**).

##### 3.3.1.2 Operative blood loss

There was no obvious heterogeneity (I^2^ = 0%, χ^2^testp=0.71) in the four studies included. A fixed effects model was adopted, and the results showed that IPA had less blood loss than EPA [OR=−20.32, 95% CI (−27.98–−12.65), p<0.00001] (**Figure 2**).

#### 3.3.2 Postoperative complications

##### 3.3.2.1 Overall complications

**Figure 3:**
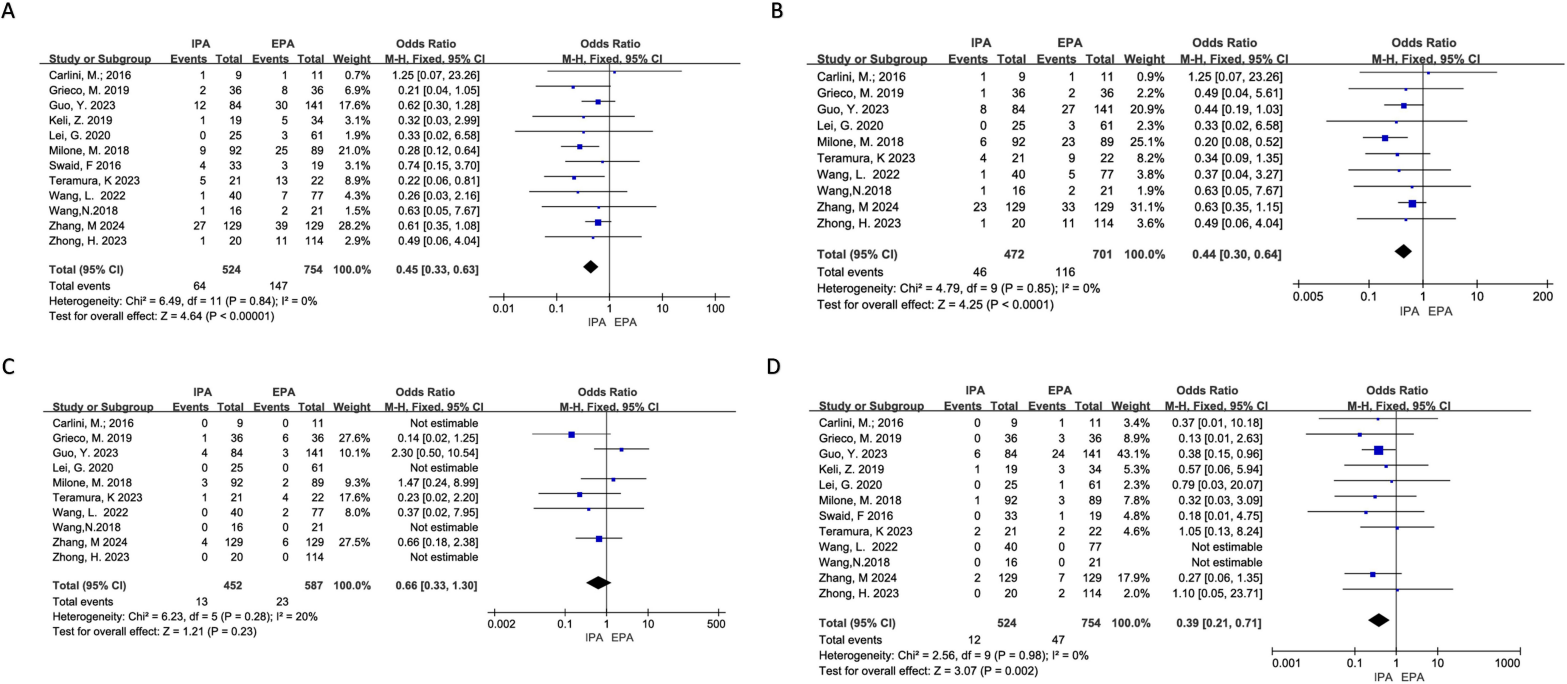
Postoperative Complications **(A)** overall Complications; **(B)** Non-severe Complications; **(C)** Severe Complications; **(D)** Surgical Site Infection.

Twelve studies reported postoperative complications. There was no heterogeneity (I^2^ = 0%, χ^2^testp=0.84). A fixed-effects model was used. The results showed that the overall complications of IPA and EPA in LLC were low, and the difference was statistically significant [OR=0.45, 95% Cl (0.33–0.63), p<0.00001] (**Figure 3**).

##### 3.3.2.2 Non-severe complications

Ten studies reported the incidence of non-severe complications (Clavien-Dindo I or II), and the heterogeneity was low (I^2^ = 0%, χ^2^testp=0.85) using a fixed-effects model. Meta-analysis results showed that the incidence of short-term non-severe complications in IPA was statistically lower than that in EPA [OR=0.44, 95% CI (0.30–0.64), p<0.0001] (**Figure 3**).

##### 3.3.2.3 Severe complications

Ten studies reported severe complications (above grade III in Clavien-Dindo), of which four did not have severe complications in both groups. Additionally, the heterogeneity was low (I^2^ = 20%, χ^2^test p=0.28). Using a fixed effects model, the results showed that there was no significant difference in the incidence of short-term severe complications between IPA and EPA [OR=0.66, 95% CI (0.33–1.30), p=0.23] (**Figure 3**).

##### 3.3.2.4 Anastomotic leakage

**Figure 4:**
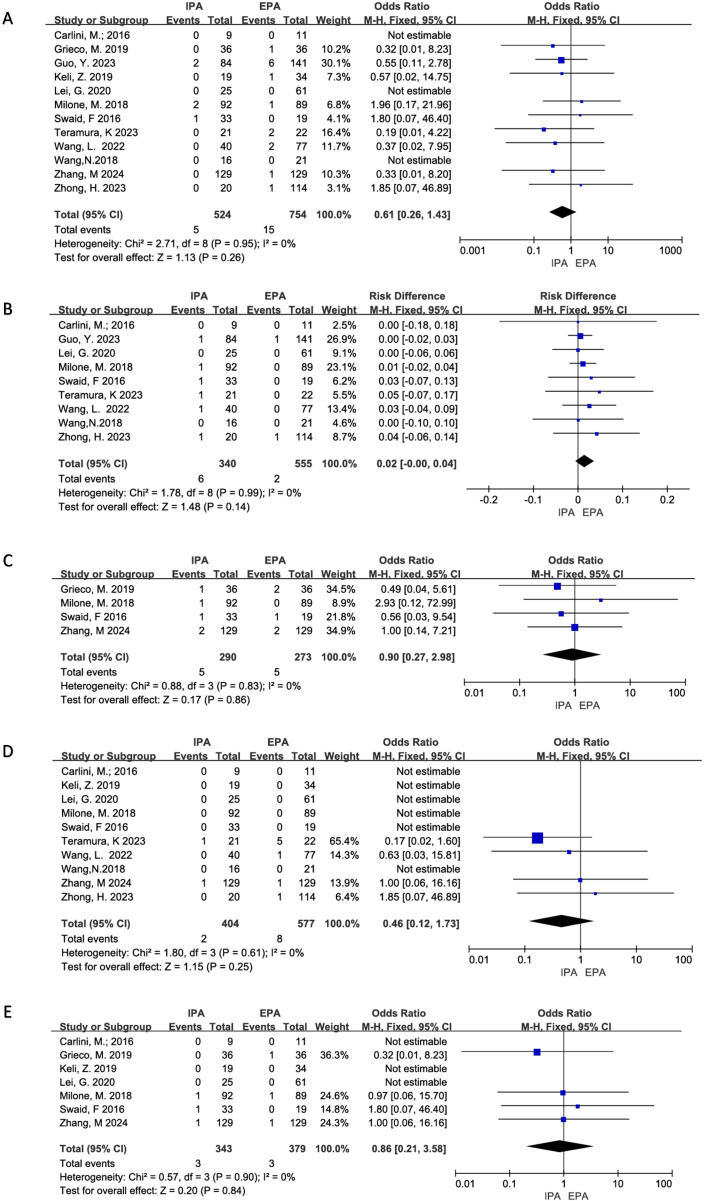
Postoperative Complications **(A)** Anastomotic Leakage; **(B)** Anastomotic Hemorrhage; **(C)** Transfusion; **(D)** Ileus; **(E)** Reoperation.

All 12 studies clearly reported the incidence of anastomotic leakage. Three studies did not have anastomotic leakage. There was no significant heterogeneity (I^2^ = 0%, χ^2^test p=0.95), and no significant difference in the incidence of anastomotic leakage between IPA and EPA using a fixed effect model [OR=0.61, 95%CI (0.26–1.43), p=0.26] (**Figure 4**).

##### 3.3.2.5 Anastomotic bleeding

Six project studies were included. There was no apparent heterogeneity (I^2^ = 0%, χ^2^testp=0.99). A fixed effects model was used, and there was no statistical difference in anastomotic bleeding between IPA and EPA [OR=0.02, 95% CI (−0.00–0.04), p=0.14] (**Figure 4**).

##### 3.3.2.6 Surgical site infections

Twelve studies were included for meta-analysis, of which two had no SSIs, and there was no significant heterogeneity (I^2^ = 0%, χ^2^testp=0.98). The results showed that the incidence of short-term incision infection in IPA was statistically lower than that in EPA using a fixed effect model [OR=0.39, 95% CI (0.21–0.71), p=0.002] (**Figure 3**).

##### 3.3.2.7 Postoperative blood transfusion

Six project studies were included for meta-analysis. There was no obvious heterogeneity (I^2^ = 0%, χ^2^testp=0.83) and no statistical difference between IPA and EPA in postoperative blood transfusion using a fixed effect model. [OR=0.90, 95%CI (0.27–2.98), p=0.86] (**Figure 4**).

##### 3.3.2.8 Postoperative ileus

Ten studies were included for meta-analysis, of which six had no intestinal obstruction and no significant heterogeneity (I^2^ = 0%, χ^2^test, p=0.61), using a fixed effect model. Meta-analysis results showed that there was no difference in postoperative ileus between IPA and EPA [OR=0.46, 95% CI (0.12–1.73), p=0.25] (**Figure 4**).

##### 3.3.2.9 Reoperation rate

Seven studies were included for meta-analysis, three of which had no reoperation and no significant heterogeneity among the studies (I^2^ = 0%, χ^2^testp=0.90). The results showed that there was no difference in postoperative intestinal obstruction between IPA and EPA using a fixed effect model [OR=0.86, 95% CI (0.21–3.58), p=0.84] (**Figure 4**).

#### 3.3.3 Oncological outcomes

##### 3.3.3.1 Number of lymph nodes dissected

**Figure 5:**
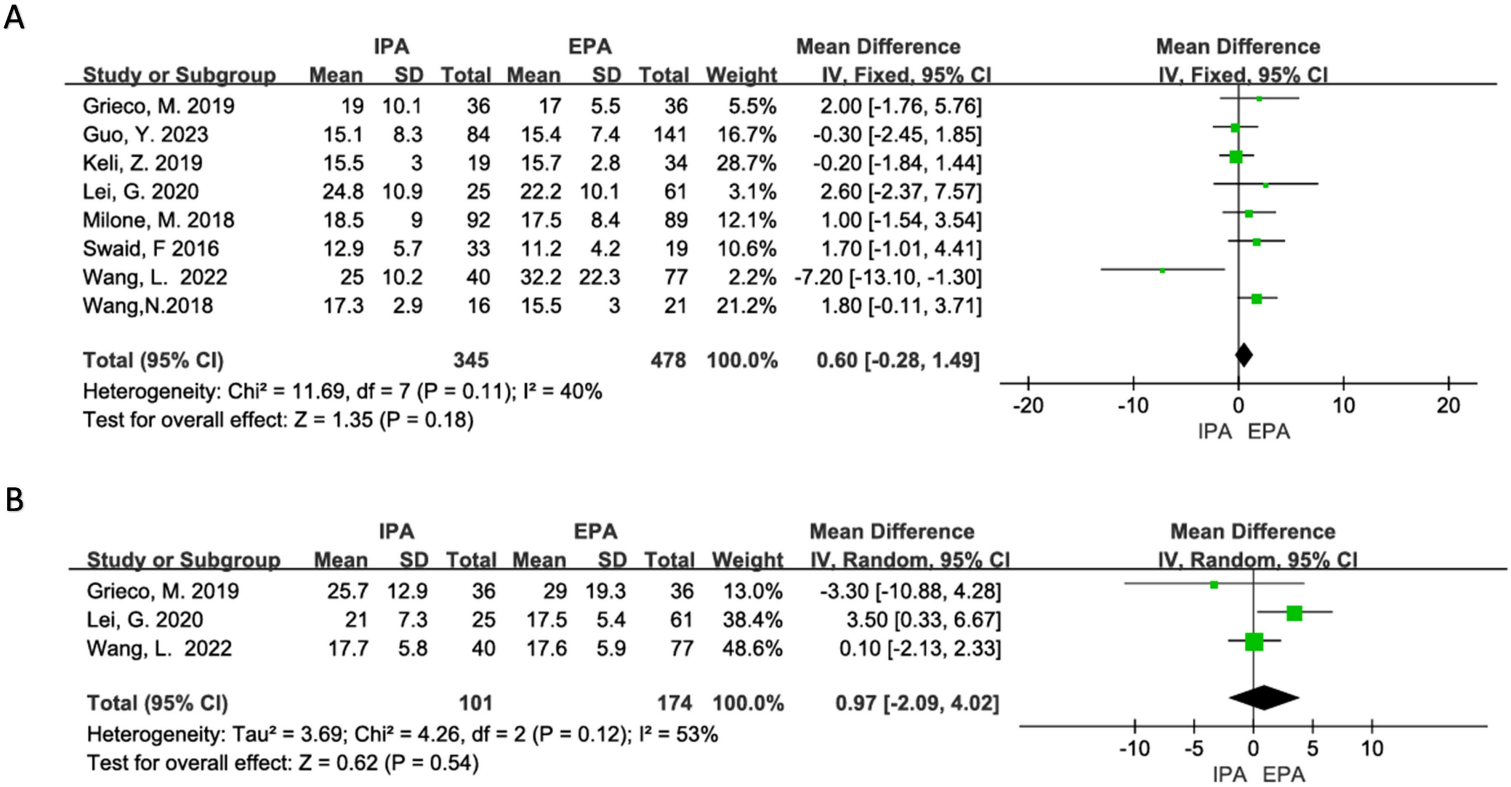
Curative effect of malignant tumor **(A)** Number of lymph node dissection; **(B)** Pathological specimen length.

Eight studies were included for meta-analysis. The heterogeneity was low (I^2^ = 40%, χ^2^testp=0.11). Using a fixed effect model, the results showed that there was no significant difference in the number of lymph node dissections between IPA and EPA in LLC [OR=0.6, 95% CI (−0.28–1.49), p=0.18] (**Figure 5**).

##### 3.3.3.2 Length of specimen

Three studies were included in the meta-analysis, but there was moderate heterogeneity (I^2^ = 53%, χ^2^testp=0.12). Sensitivity analysis suggested that Lei, G’s. 2020 studies may be the source of heterogeneity, but there was no clinical reason to exclude it from the meta-analysis. Therefore, the random-effects model was used, and it showed that there was no difference in the length of the specimen between IPA and EPA [OR=0.97, 95% CI (−2.09–4.02), p=0.54] (**Figure 5**).

#### 3.3.4 Postoperative rehabilitation

##### 3.3.4.1 Time to flatus

**Figure 6:**
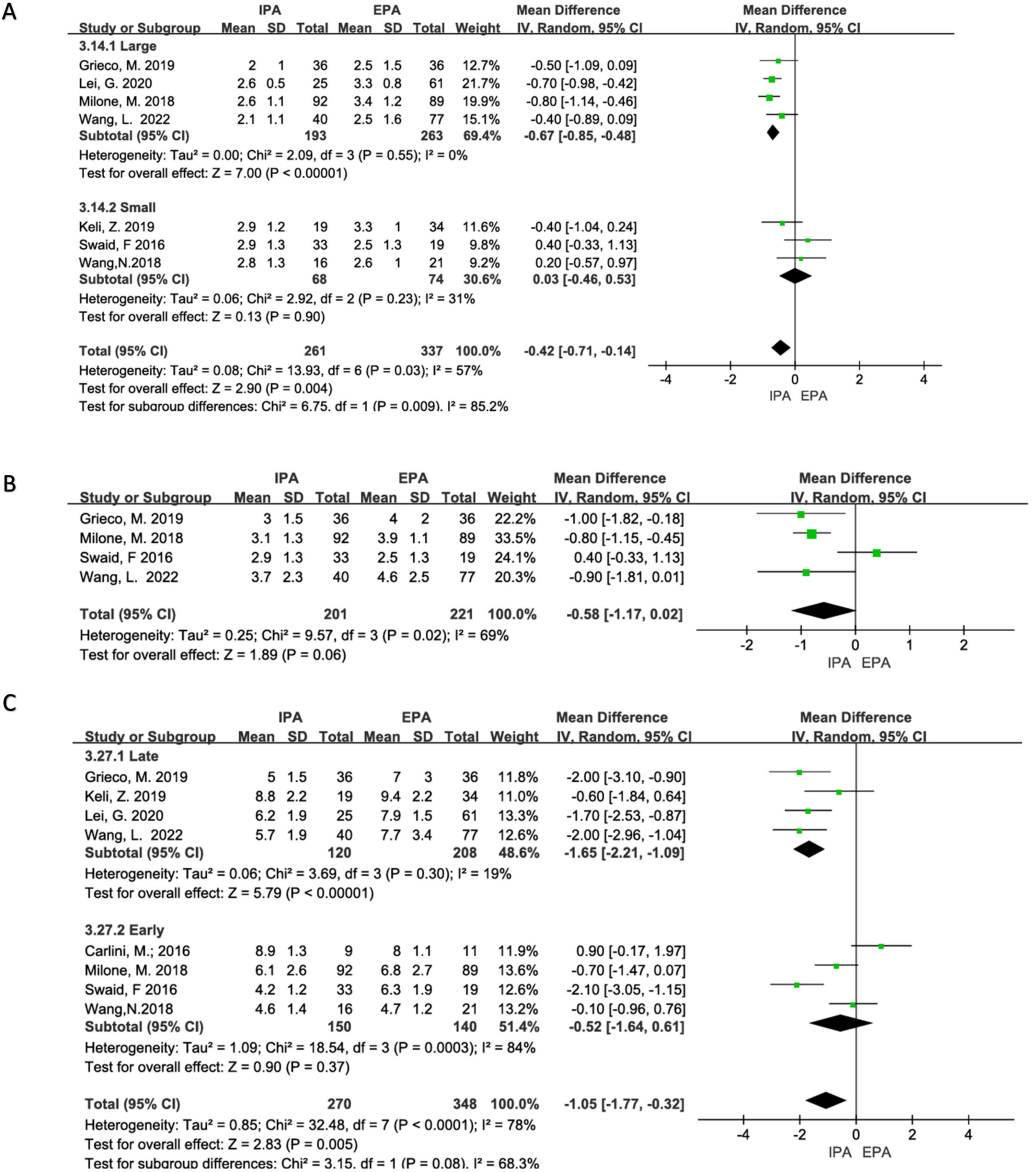
Status of postoperative recovery **(A)** Time to first flatus; **(B)** Time to first stool; **(C)** Length of hospital stays.

Seven studies were included with a moderate heterogeneity (I^2^ = 57%, χ^2^testp=0.03). Using a random-effects model, the results showed that the time to flatus of IPA was statistically shorter than that of EPA [OR=−0.42, 95% CI (−0.71–−0.14), p=0.004]. Heterogeneity decreased in subgroup analysis (large sample group, I^2^ = 0%, χ^2^testp=0.55; small sample group, I^2^ = 31%, χ^2^testp=0.23), and the time to flatus of IPA in the large sample subgroup was shorter than that of EPA [OR=−0.67, 95%CI (−0.85–−0.48), p<0.00001], and there was no statistical difference in the small sample subgroup [OR=0.03, 95% CI (−0.46–0.53), p=0.9] (**Figure 6**).

##### 3.3.4.2 Time to stool

Four studies were included in the analysis, and there was moderate heterogeneity (I^2^ = 69%, χ^2^testp=0.02). Therefore, a random-effects model was used in the meta-analysis. The results did not show a statistical difference between the two groups. [OR=−0.58, 95% CI (−1.17 –−0.02), p=0.06] (**Figure 6**). A sensitivity analysis was performed, and Swaid, F may be a source of heterogeneity due to the small sample size, but there was no sufficient clinical reason to exclude it.

##### 3.3.4.3 Length of hospital stays

Eight studies were included in the analysis with obvious heterogeneity (I^2^ = 78%, χ^2^testp<0.0001). Sensitivity analysis could not find the source of the heterogeneity. Therefore, a random-effects model was used. The results showed that the length of hospital stays of IPA was statistically shorter than that of EPA [OR =−1.05, 95% CI (−1.77–−0.32), p=0.005]. In subgroup analyses, studies published after 2019 had a reduced heterogeneity (I^2^ = 19%, Chi-square test p=0.30), and hospital stays were statistically shorter for IPA than for EPA [OR=−1.65, 95% CI (−2.21–−1.09), p<0.00001] (**Figure 6**).

### 3.4 Publication bias evaluation

**Figure 7:**
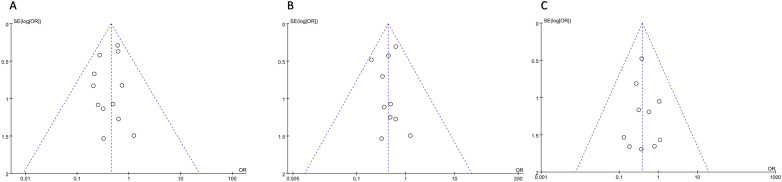
Postoperative Complications **(A)** overall Complications; **(B)** Non-severe Complications; **(C)** Surgical Site Infection.

Funnel maps were plotted based on overall postoperative complications, non-severe complications, and SSIs for IPA and EPA. The funnel plot shows a symmetrical distribution of studies, which means there was no significant publication bias in this meta-analysis (**Figure 7**).

## 4 Discussion

Laparoscopic colectomy, first reported by Jacobs in 1991, has received high attention from surgeons due to its minimally invasive advantages (30). With the improvement in surgeons’ laparoscopic skills and the development of laparoscopic instruments and robots, some surgeons began to explore more extreme minimally invasive surgery (31–33). In recent years, IPA in laparoscopic right colectomy has been widely recognized (34). However, unlike ileo-colon anastomosis in right colectomy, colic-colon anastomosis in left colectomy requires a longer mobilized colon to achieve a tension-free anastomosis. Therefore, the difficulty of colon resection and anastomosis in the left colectomy is more significant than that in the right colectomy (20, 35, 36).

The surgical modalities for left colon cancer are still controversial (37–39). Owing to the low morbidity, previous studies comparing the difference between IPA and EPA in left colectomy were mostly small sample cohort studies. Our study comprehensively evaluated the differences between IPA and EPA from four aspects: difficulty of surgery, postoperative complications, postoperative rehabilitation, and oncological outcomes. Owing to the significant difference between transanal IPA and transabdominal IPA, this study excluded the study of transanal IPA. Compared with a previous study (7), our study includes more articles and a larger sample size. Although the short-term complications and postoperative recovery were partially similar to those in the previous study, we conducted a detailed subgroup analysis and evaluated the difficulty of surgery and the oncological outcomes. We also performed a publication bias analysis, which suggested that the results of our meta-analysis were stable.

Regarding surgical difficulty, the meta-analysis results suggested that IPA had less bleeding than EPA. Although there was no statistical difference in operative time between the two groups, after subgroup analysis, we found that IPA in the multicenter subgroup and the PSM study subgroup had longer operative times than the EPA group, with low heterogeneity. This may mean that IPA is more complex and challenging to operate, which is consistent with our clinical experience (there are no specific studies). Intraoperative complications were reported in three studies, all of which occurred in the *in vitro* anastomosis group, including splenic injury (29), small intestine injury (28), and abdominal hemorrhage (21), which may be because anastomosis in the abdominal cavity requires the mobilization of a longer colon and thus consumes more time. There was no meta-analysis performed on conversion to open surgery as only two studies had positive reports about it. In addition, two studies did not include cases of conversion to open surgery.

In this study, we also found that the reduction of overall complications in IPA was mainly due to a decrease in non-severe complications, which was not suggested by the previous study. Although IPA was more difficult to carry out, there was no statistical difference in anastomose-related complications between the two groups, which may also be due to an insufficient sample size. Although the SSI of IPA is reduced when compared with EPA, there is insufficient literature to evaluate whether this reduction is due to superficial, deep, or organ infection. However, this is very important as infection at different surgical sites affects patients differently (40, 41), and this may be a potential research direction. The incision length is generally shorter in IPA (**Table 3**), which may be one reason for the fewer SSIs in IPA. Different extraction sites may affect the incidence of incisional infection and incisional hernia, which was the conclusion in previous studies (42, 43). However, too many extraction sites were involved in the 12 studies our study included; therefore, it is difficult to compare the specificity of the different extraction sites directly. Regarding long-term complications, only Grieco, M. reported more incisional hernias in the EPA group than in the IPA group (24). Meta-analysis was not performed for some less reported complications, such as abdominal hemorrhage, which was only reported in two studies (19, 27).

Regarding the oncological outcomes, the number of lymph nodes dissected and the length of specimen resected showed no statistical difference between the two groups, which was consistent with that in right colectomy (44) but inconsistent in gastric cancer surgery (27, 45). In fact, the length between the tumor and the resection margin is more significant than the specimen length. However, only four studies reported the length between the tumor and the resection margin. The reported forms were different, including the average resection margin length (21), the nearest resection margin length (22), and the length between the tumor and the distal or proximal resection margins (13) (19); therefore, it was not possible to conduct a meta-analysis on resection margin length. Among all the studies, only Zhong H reported the incidence of resection margin length deficiency, and intraperitoneal anastomosis had a lower incidence of resection margin length deficiency than EPA (21). However, the short-term oncological outcomes do not necessarily reflect the long-term survival difference because the surgical method and the scope of lymph node dissection for splenic flexural tumors are still controversial (37, 39, 46). In this study, the long-term oncological outcome indicators reported by different studies were also different. Three studies reported the differences in long-term recurrence and metastasis between the two groups (19, 22, 29), 2-year overall survival (OS) and disease-free survival (DFS) (22) and 5-year OS and DFS (19), but the results were not statistically significant. None of the studies reported excision of the mesangial area or number of lymph nodes at different sites. In general, the limited indicators in this study suggest that the short-term oncological outcomes of the two anastomoses are consistent. However, the long-term oncological outcomes need more data to test.

When conducting meta-analyses of postoperative rehabilitation-related indicators, we found that the heterogeneity of the studies was high, and more reliable results may be obtained through subgroup analysis. Different extraction sites have different effects on abdominal nerves and muscles, which may affect the speed of postoperative rehabilitation. We divided these extraction sites into three categories, Pfannenstiel, longitudinal midline incision, and various types of non-midline incision, as there are too many extraction sites, and it is impossible to directly compare the influence of different extraction sites on postoperative recovery. Even so, subgroup analysis suggested that extraction sites were not the source of heterogeneity. Overall, only the large sample subgroup and the subgroup of recently published studies had low heterogeneity. The IPA group in these subgroups had an earlier exhaust time and shorter hospital stays. Only two studies reported the time spent on oral eating and time spent on free movement after surgery; therefore, a meta-analysis was not performed. Meta-analysis could not be performed in the evaluation of postoperative pain either because only one study applied the NSC pain score for four consecutive days after surgery (22), two studies used salvage analgesics (13, 19), and one study evaluated postoperative pain perception through morphine use (22). Although there were three studies with VAS scores (13, 25, 27), the literature did not mention the time of VAS evaluation.

This study also has other obvious limitations as follows: (1) the included studies were all cohort studies, lacking multicenter, large sample, and PSM studies; (2) unpublished clinical studies were not included, with potential publication bias; (3) meta-analysis of long-term complications and oncological outcomes was impossible due to insufficient literature data; and (4) much of the literature did not provide data on prophylactic antibiotics, intestinal preparation, ERAS, etc, therefore, there is some interference in evaluating the speed of postoperative recovery and the incidence of SSI.

## 5 Conclusion

Intraperitoneal anastomosis in laparoscopic left colectomy has a lower complication rate, faster postoperative recovery, and no short-term oncological outcome difference compared with extraperitoneal anastomosis. However, it may be more complex and takes more time to carry out, and at the same time, the long-term complications and oncological outcomes are still uncertain. Therefore, the results of this study need to be verified by a large multicenter randomized controlled trial.

## Data Availability

The original contributions presented in the study are included in the article/**Supplementary Material**. Further inquiries can be directed to the corresponding authors.
