# Supplementary figures and images for: A systematic review and meta-analysis of intraperitoneal anastomosis versus extraperitoneal anastomosis in laparoscopic left colectomy
Source: Front Oncol. 2024 Sep 27;14:1464758. doi: 10.3389/fonc.2024.1464758 (PMC11466934; doi:10.3389/fonc.2024.1464758)

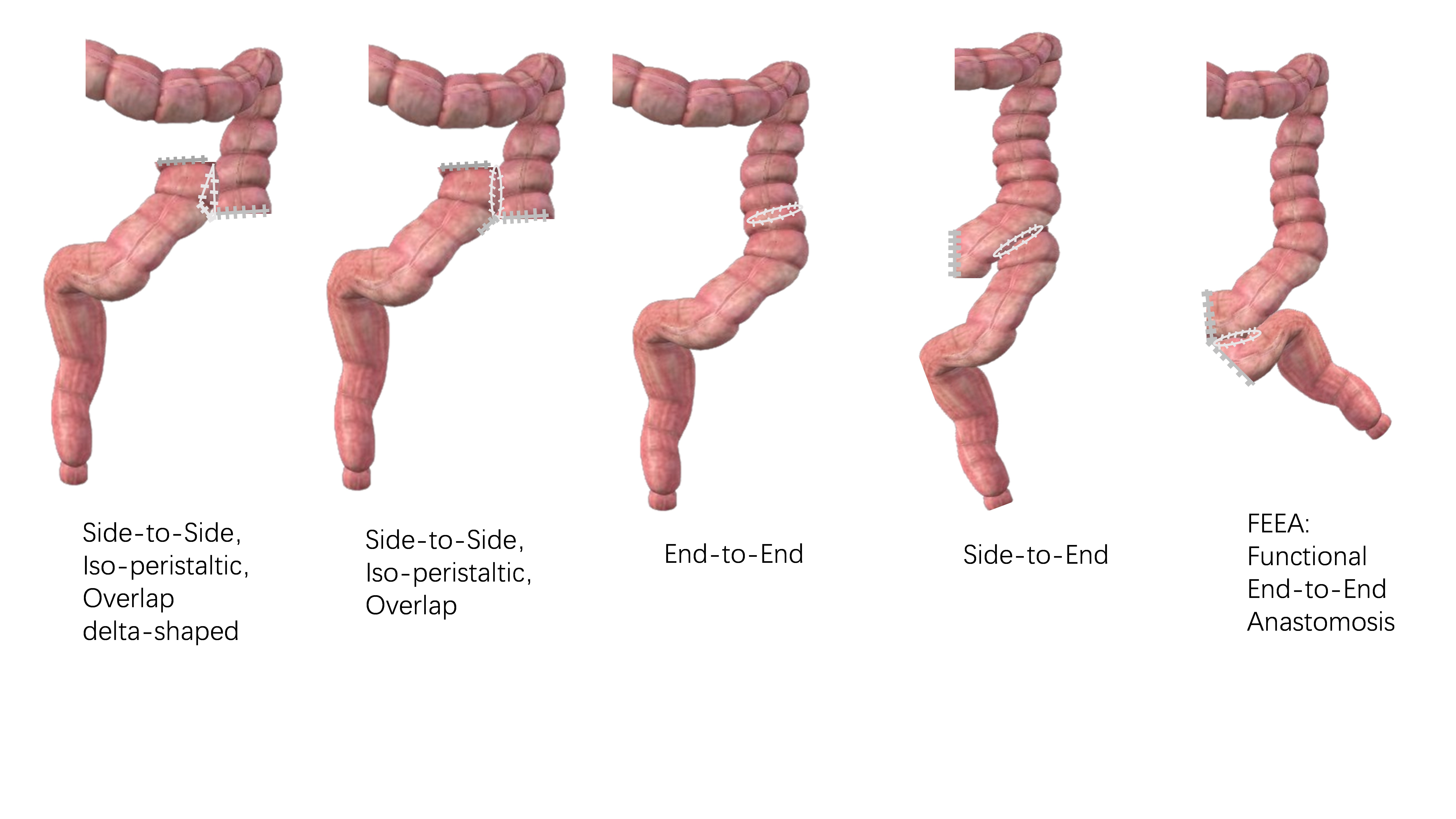

Supplement: Supplementary file 1 [file Image1.jpeg]
